# Four-Dimensional Continuity Gaps in Volunteer-Integrated Services for Older Adults: Mixed-Methods Study in China

**DOI:** 10.1093/geroni/igaf122.3937

**Published:** 2025-12-31

**Authors:** Mingzhu Yang, Shuang Cai, Jinsong Pan

**Affiliations:** Shanghai General Hospital Shanghai Jiao Tong University School of Medicine, Baltimore, Maryland, United States; Shanghai General Hospital Shanghai Jiao Tong University School of Medicine, Shanghai General Hospital, Shanghai Jiao Tong University School of Medicine, Shanghai, China (People’s Republic); Shanghai General Hospital, Shanghai Jiao Tong University School of Medicine, Shanghai, China (People’s Republic)

## Abstract

Rapid urban population ageing in China is increasing demand for community-based services for older adults. Volunteer-supported programs, offering companionship, emotional support, and assistance with daily tasks, are promoted as complements to formal care, yet their integration into resilient systems with functional, relational, informational, and managerial continuity remains unclear. This study aimed to identify barriers, examine service discontinuities, and integrate findings within a four-dimensional continuity framework to inform strategies for cohesive volunteer engagement. An explanatory sequential mixed-methods design was applied in districts with the highest proportion of older adults, Hongkou and Huangpu, Shanghai. A cross-sectional survey of 880 older adults (response rate 92.9%) measured willingness, utilization, and predictors of underuse or dissatisfaction. Multivariable logistic regression, adjusted for demographic and health factors, identified predictors. In-depth interviews with 21 older adults, volunteers, and Red Cross staff explored mechanisms. Findings were integrated via the Pillar Integration Process, mapping to four continuity domains. Although 96.3% expressed willingness, only 46.5% used services in the past year. Digital illiteracy, particularly among those aged > =80 years (89.3%), and high volunteer turnover ( > =3 changes in 6 months) significantly predicted underuse and dissatisfaction (adjusted odds ratio=2.31). Qualitative results revealed policy–service dissonance shifting unmet needs to volunteers, relational instability from turnover, digital–informational gaps causing fragmented handovers, and governance fragmentation marked by absent procedures and siloed coordination. Bridging this gap requires expanding Long-Term Care Insurance to moderately disabled adults, embedding volunteers in stable interdisciplinary teams, introducing care navigation for unmet needs, and improving digital literacy via hybrid record systems.

